# Cellular and Molecular Mechanisms of Liver Fibrosis in Patients with NAFLD

**DOI:** 10.3390/cancers15112871

**Published:** 2023-05-23

**Authors:** Jessica I. Sanchez, Edwin R. Parra, Jingjing Jiao, Luisa M. Solis Soto, Debora A. Ledesma, Omar A. Saldarriaga, Heather L. Stevenson, Laura Beretta

**Affiliations:** 1 Department of Molecular and Cellular Oncology, The University of Texas MD Anderson Cancer Center, Houston, TX 77030, USA; 2 Department of Translational Molecular Pathology, The University of Texas MD Anderson Cancer Center, Houston, TX 77030, USA; 3 Department of Pathology, The University of Texas Medical Branch, Galveston, TX 77555, USA

**Keywords:** NAFLD, liver fibrosis, multiplexed imaging technologies

## Abstract

**Simple Summary:**

Nonalcoholic fatty liver disease (NAFLD) is rapidly becoming a major cause of cirrhosis and hepatocellular carcinoma (HCC). The risk of liver-related mortality, including HCC, significantly increases with the progression of liver fibrosis in NAFLD patients. This study aimed to characterize the molecular and cellular changes occurring during liver fibrosis progression in NAFLD patients. The identified gene and immune cell signatures may lead to novel surveillance and prevention strategies.

**Abstract:**

The expression of immune- and cancer-related genes was measured in liver biopsies from 107 NAFLD patients. The strongest difference in overall gene expression was between liver fibrosis stages F3 and F4, with 162 cirrhosis-associated genes identified. Strong correlations with fibrosis progression from F1 to F4 were observed for 91 genes, including CCL21, CCL2, CXCL6, and CCL19. In addition, the expression of 21 genes was associated with fast progression to F3/F4 in an independent group of eight NAFLD patients. These included the four chemokines, SPP1, HAMP, CXCL2, and IL-8. A six-gene signature including SOX9, THY-1, and CD3D had the highest performance detecting the progressors among F1/F2 NAFLD patients. We also characterized immune cell changes using multiplex immunofluorescence platforms. Fibrotic areas were strongly enriched in CD3^+^ T cells compared to CD68^+^ macrophages. While the number of CD68^+^ macrophages increased with fibrosis severity, the increase in CD3^+^ T-cell density was more substantial and progressive from F1 to F4. The strongest correlation with fibrosis progression was observed for CD3^+^CD45R0^+^ memory T cells, while the most significant increase in density between F1/F2 and F3/F4 was for CD3^+^CD45RO^+^FOXP3^+^CD8^−^ and CD3^+^CD45RO^−^FOXP3^+^CD8^−^ regulatory T cells. A specific increase in the density of CD68^+^CD11b^+^ Kupffer cells with liver fibrosis progression was also observed.

## 1. Introduction

Nonalcoholic fatty liver disease (NAFLD) is the most common liver disease worldwide, with an increase in prevalence in the United States (US) from 20.0% in the years 1988–1994 to 31.9% in the years 2013–2016 [1,2]. It was estimated that there were 64 million people with NAFLD in the US in 2018 [3]. Nonalcoholic steatohepatitis (NASH), the most severe form of NAFLD, is now the second most common indication for liver transplantation and is rapidly becoming a major cause of cirrhosis and hepatocellular carcinoma (HCC) [4,5]. The prevalence of both NAFLD and NASH is expected to continue to increase by 21% and 63%, respectively, by 2030 [6]. The risk of liver-related mortality, including HCC, significantly increases with the progression of liver fibrosis in NAFLD patients [7,8]. Although there are several drugs in development for NASH and liver fibrosis therapies, none has been approved to date [9]. Identifying the molecular and cellular mechanisms that contribute to liver fibrosis progression in NAFLD patients may lead to novel surveillance and prevention strategies.

A few transcriptomic profiling studies in NAFLD have been reported in recent years. Because there are no reliable non-invasive diagnostics for distinguishing NASH from NAFLD, a major focus of these studies was to identify signatures that distinguish NASH in NAFLD patients [10,11,12]. These studies showed that the functions of the transcriptomic changes in NASH development were largely associated with changes in liver fibrosis severity. Some studies identified fibrosis-specific signatures in NAFLD [13,14,15]. In addition, single-cell transcriptomic studies have been performed, but they largely focused on hepatic stellate cells (HSCs), identifying distinct populations of activated HSCs associated with the level of fibrosis [16] as well as master regulators responsible for the activation of HSCs during fibrogenesis [14,17]. The cell-type composition deconvolution of fibrosis in NAFLD suggested, however, that single-cell transcriptomic studies should be performed in additional cell types such as hepatocytes and progenitor cells [15,18].

In this study, we combined targeted liver gene expression profiles, predictive cell population and pathway analysis, and multiplex immunofluorescence staining for the spatial distribution of selected immune cell types to characterize molecular and cellular changes occurring between liver fibrosis stages F4 and F3 as well as during the progression of liver fibrosis from stage F1 to F4. We further delineated the gene expression signature that best identified patients with F1/F2 who rapidly progressed to F3/F4. Altogether, we identified the key genes and molecular mechanisms of fibrosis progression in NAFLD. Such signatures could serve as predictive markers or preventive therapeutic targets.

## 2. Materials and Methods

### 2.1. Patients and Liver Biopsy Samples

The study was approved by the Committee for the Protection of Human Subjects of The University of Texas Medical Branch and The University of Texas MD Anderson Cancer Center. Archived formalin-fixed paraffin-embedded (FFPE) liver biopsies from 99 patients with NAFLD, obtained between 2006 and 2019 as standard of care at The University of Texas Medical Branch through the percutaneous route using an 18-gauge core needle, were employed for this study. An additional set of FFPE liver biopsies from eight NAFLD patients with liver fibrosis stages F1 or F2 was obtained. These patients were identified retrospectively, as fast progressors, based on progression to F3 or F4 within 10 years. Demographic and clinical parameters for all patients are shown in Supplementary Table S1. At collection, biopsies were immediately placed into 10% buffered formalin; processed using a TissueTekVIP tissue processor (Sakura Finetek, Torrance, CA, USA); and paraffin-embedded. Each patient’s biopsy was evaluated by Dr. Stevenson, liver pathologist, using the criteria reported by Brunt et al. [19] for the stage of fibrosis (0: no fibrosis; 1: mild/moderate zone-three perisinusoidal fibrosis, or portal/periportal fibrosis only; 2: perisinusoidal and portal/periportal fibrosis; 3: bridging fibrosis; 4: cirrhosis) and components of NAFLD activity score (NAS)—steatosis (S0: <5%, S1: 5–33%, S2: >33–66%, S3: >66%); lobular inflammation (I0: no foci, I1: <2 foci per 200x field, I2: 2–4 foci per 200x field, I3: >4 foci per 200x field); and hepatocyte ballooning (B0: none, B1: few ballooning cells, B2: many cells with prominent ballooning).

### 2.2. RNA Isolation and Targeted Gene Expression Profiling

FFPE tissue blocks were sectioned to obtain 2 to 3 sections at a thickness of 3 µm. RNA was extracted using a High Pure FFPET RNA Isolation kit (Roche), its concentration was measured with Qubit fluorometer (Life Technologies, Carlsbad, CA, USA), and quality control was performed on a bioanalyzer using an RNA6000 pico assay. The percentage of RNA fragments above 200 nucleotides was used to adjust the RNA input. Gene expression was investigated using the PanCancer Immune Profiling and PanCancer Pathways panels (NanoString Technologies, Seattle, WA, USA) on the nCounter^®^ SPRINT platform. Gene expression data were analyzed using NanoString’s software nSolver V.4.0 with the Advanced Analysis 2.0 plugin. Data were normalized using the Advanced Analysis tool, which draws on the NormqPCR R package [20]. Statistically significant, differentially expressed genes between two groups were defined as fold change (FC) ≥1.5 or ≤−1.5 and *p* ≤ 0.05. To control for the false discovery rate, *p* values were also adjusted using the Benjamini–Hochberg method. Immune cell and pathway analyses were performed using NanoString’s advanced analysis module [21]. In brief, immune cell scores were calculated using marker genes expressed stably and specifically in immune cell types, and estimated relative abundances were measured as the average log-scale expression of their characteristic genes. Pathway scores were calculated as the first principal component of the pathway genes’ normalized expression [22]. Core analyses of differentially expressed genes were also performed using the Ingenuity Pathway Analysis platform (Qiagen, Germantown, MD, USA).

### 2.3. Multiplex Immunofluorescence (mIF) and Digital Image Analysis

Two mIF panels were used to characterize the immune microenvironment in 22 selected NAFLD liver biopsies (11 per panel). The staining was performed using similar methods and reagents to those that have been previously described [23]. The distribution of fibrosis stages among the selected 11 biopsies per panel were as follows: three F1, three F2, two F3, and three F4. For staining, 4 µm thick FFPE liver tissue sections were stained using the automated staining system Leica BOND-RX (Leica Microsystems, Buffalo Grove, IL, USA). Panel 1 included antibodies against CD3 (clone D7A6E, dilution 1:100, Cell Signaling Technology, Danvers, MA, USA); CD8 (clone C8/144B, dilution 1:25, Dako, Santa Clara, CA, USA); CD45RO (clone UCHL1, Leica Microsystems, Buffalo Grove, IL, USA); FOXP3 (clone D2W8E, dilution 1:50, Cell Signaling Technology, Danvers, MA, USA); PD-1 (clone EPR4877(2), dilution 1:250, Abcam, Cambridge, MA, USA); and CD68 (clone PG-M1, dilution 1:50, Dako, Santa Clara, CA, USA). Panel 2 included antibodies against Arg-1 (clone D4E3M, dilution 1:250, Cell Signaling Technology, Danvers, MA, USA); CD11b (clone EPR1344, dilution 1:6000, Abcam, Cambridge, MA, USA); CD14 (clone SP192, dilution 1:300, Abcam, Cambridge, MA, USA); CD33 (clone PWS44 (M), dilution 1:50, Leica Microsystems, Buffalo Grove, IL, USA); CD66b (clone G10F5, dilution 1:100, BioLegend, San Diego, CA, USA); and CD68 (clone PG-M1, dilution 1:50, Dako, Santa Clara, CA, USA). All antibodies were linked with one of the fluorophores from the Opal 7 IHC kit (catalog no. NEL797001KT; Akoya Biosciences, Waltham, MA, USA) and the TSA fluorophore Opal Polaris 480 (#FP1500001KT, Akoya Biosciences, Waltham, MA, USA). Stained slides were scanned using a multispectral microscope (PhenoImager 1.0.13 imaging system, Akoya Biosciences, Waltham, MA, USA) under fluorescence and low magnification at 10x. Following scanning, a pathologist selected representative regions of interest (each ROI, 0.63 mm^2^) per sample from fibrotic areas (with an accumulation of excess extracellular matrix components) using the phenochart 1.0.9 viewer (Akoya Biosciences, Waltham, MA, USA) guided by the H&E-stained histopathologic features. Imaging analysis was performed with quantitative image analysis software (InForm, Akoya Biosciences, Waltham, MA, USA) using spectral libraries defined with single-marker immunofluorescence detection. Marker colocalization was employed to identify different cellular phenotypes and quantified as the number of cells/mm^2^ on fibrotic areas and transitions with the normal liver tissue compartment. Data were consolidated using R studio 3.5.3 (Phenopter 0.2.2 packet, Akoya Biosciences, Waltham, MA, USA).

### 2.4. Statistical Analysis

Principal component analyses (PCoAs) were performed with Euclidian-based distance matrices generated in R using log2-transformed gene expression values and log10-transformed expression values alongside the permutational multivariate analysis of variance (PERMANOVA) test for statistical significance. Volcano plots, box plots, and heatmaps were generated in GraphPad Prism 8.0.0. The pROC package in R was used to generate receiver operating characteristic (ROC) curves and compute AUC values with 95% confidence intervals (CIs). The corrplot package in R was used to generate the correlation matrix.

## 3. Results

### 3.1. Characteristics of the NAFLD Patient Cohort

A total of 99 patients with liver-biopsy-proven NAFLD were included in this study. The demographic and clinical parameters of these NAFLD patients are summarized in Supplementary Table S1. Overall, the patients were predominantly females (67%), and the median age was 50 years old. The majority of subjects were obese (87%), with a median BMI of 42.4. Type 2 diabetes was also prevalent (51%). The majority of subjects were White (74% non-Hispanic and 18% Hispanic). The distribution of liver fibrosis stages was 32% F1, 30% F2, 15% F3, and 22% F4. The distribution of steatosis was 36% with mild steatosis (S1), 43% with moderate steatosis (S2), and 17% with marked steatosis (S3). In addition, three of the subjects had F4 fibrosis (cirrhosis) and burned-out NASH (S0). The proportion of patients with no ballooning (B0), few ballooning cells (B1), and prominent ballooning (B2) was 35%, 51%, and 14%, respectively. The proportion of patients with no lobular inflammation (I0), mild inflammation (I1), moderate inflammation (I2), and strong inflammation (I3) was 4%, 48%, 43%, and 4%, respectively. Lastly, 43 of the 99 patients (43%) had an NAFLD activity score (NAS) ≥5 and therefore a definite diagnosis of NASH.

### 3.2. Hepatic Gene Expression Changes in NAFLD Patients with Cirrhosis

**Figure 1:**
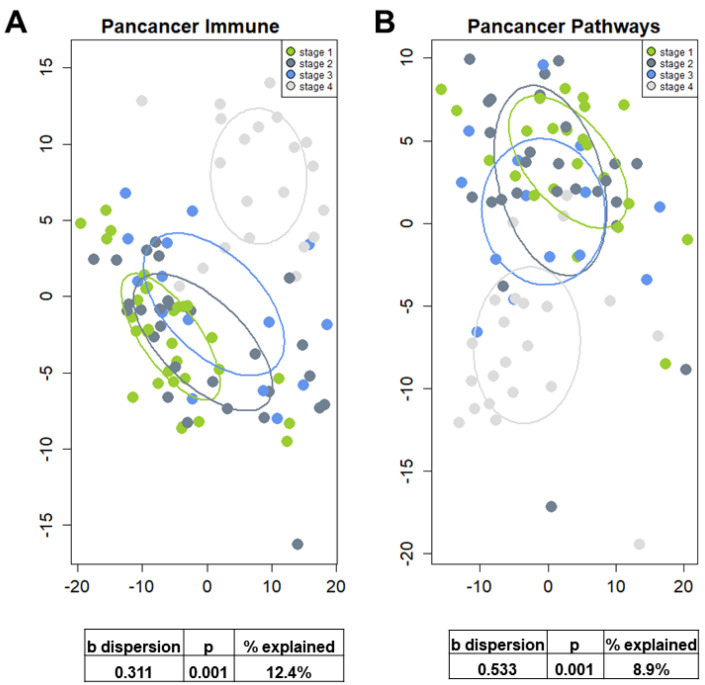
Principal component analysis (PCA) of gene expression data from PanCancer Immune Profiling panel (**A**) and PanCancer Pathways panel (**B**). Ellipses were drawn using the standard deviation of point scores. Log2-transformed gene expression data were used. Insert: Permutational multivariate analysis of variance (PERMANOVA) test results including beta dispersion, percentage of variance explained, and *p* value.

The expression of 770 immune-related genes and 770 genes related to cancer-associated canonical pathways was measured in RNA extracted from the same archived liver biopsies using two targeted panels (PanCancer Immune Profiling and PanCancer Pathways). For both panels, PCoA showed a clear separation of the samples from subjects with cirrhosis (F4), while stages F1-F3 showed a progressive shift, with 12.4% and 8.9% of the gene expression profiles explained by fibrosis stage in the PanCancer Immune Profiling panel (Figure 1A) and the PanCancer Pathways panel (Figure 1B), respectively (*p* = 0.001).

**Figure 2:**
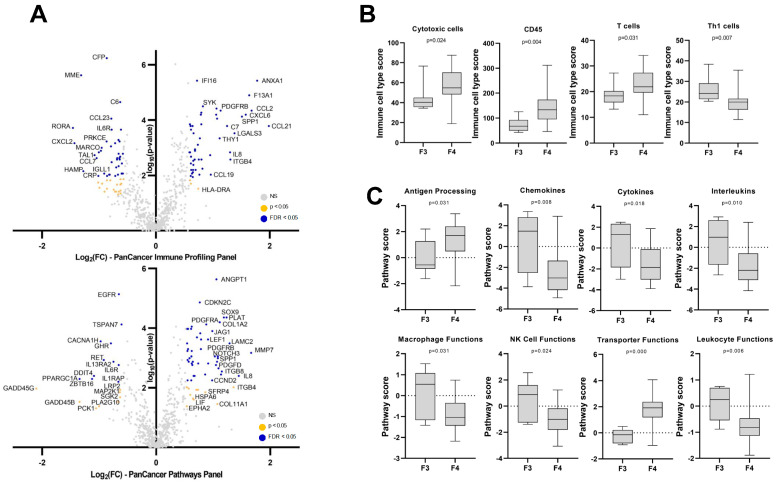
Differentially expressed genes in F4 livers compared to F3 livers. (**A**) Volcano plots showing gene expression changes using PanCancer Immune Profiling panel (**top**) and PanCancer Pathways panel (**bottom**). Log2 fold change (x-axis) and minus log10 *p*-value (y-axis) were used. Genes with FC ≥ 1.5 or ≤−1.5 and *p* ≤ 0.05 are shown in orange. Genes that remained significant after adjustment with the Benjamini–Hochberg method are shown in blue. (**B**) Box plots of immune cell type scores in F3 and F4 livers. (**C**) Box plots of pathway scores in F3 and F4 livers. Cell type and pathway scores were obtained using NanoString advanced analysis modules. *p* values in panels B and C were calculated using Mann–Whitney test.

With both panels combined, the expression of 90 genes was increased, and the expression of 72 genes was decreased in liver biopsies from patients with cirrhosis (F4) compared to patients with F3 fibrosis (Supplementary Table S2). Significance remained after adjusting for the false discovery rate (FDR) (q ≤ 0.05) for 126 of the 162 genes. The largest increases were observed for C-C or C-X-C motif chemokine ligands CCL21 (fold change (FC) = 4.0, q < 0.001); CCL2 (FC = 3.2, q < 0.001) and CXCL6 (FC = 3.0, q < 0.001); annexin A1 (ANXA1) (FC = 3.4, q < 0.001); matrix metallopeptidase 7 (MMP7) (FC = 3.2, q = 0.013); coagulation factor XIII A (F13A1) (FC = 3.1, q < 0.001); osteopontin (SPP1) (FC = 2.9, q < 0.001); interleukin 8 (IL8) (FC = 2.7, q = 0.036); galectin 3 (LGALS3) (FC = 2.6, q < 0.001); integrin subunit beta 4 (ITGB4) (FC = 2.5, q = 0.020); and laminin subunit gamma2 (LAMC2) (FC = 2.4, q < 0.001), while the largest decreases were observed for RAR-related orphan receptor A (RORA) (FC = −2.8, q < 0.001); CXCL2 (FC = −2.7, q = 0.009); PPARG coactivator 1 alpha (PPARGC1A) (FC = −2.5, q = 0.042); membrane metalloendopeptidase (MME) (FC = −2.5, q < 0.001); hepcidin antimicrobial peptide (HAMP) (FC = −2.4, q = 0.037); zinc finger and BTB domain containing 16 (ZBTB16) (FC = −2.8, q = 0.042); DNA damage inducible transcript 4 (DDIT4) (FC = −2.1, q = 0.036); TAL BHLH transcription factor 1 (TAL1) (FC = −2.1, q = 0.015); CCL7 (FC = −2.1, q = 0.015); and C-reactive protein (CRP) (FC = −2.0, q = 0.045) (Figure 2A). Using the NanoString advanced analysis module, several immune cell populations were predicted to be significantly enriched or depleted in the livers of patients with cirrhosis compared to patients with liver fibrosis F3. Notably, CD45^+^ cells (*p* = 0.004), cytotoxic cells (*p* = 0.024), and T cells (*p* = 0.031) were significantly enriched in patients with cirrhosis, while Th1 cells (*p* = 0.007) were significantly depleted (Figure 2B). The Nanostring advanced analysis module’s pathway analysis tool also identified that antigen processing (*p* = 0.031) and transporter function (*p* < 0.001) pathways were enriched, while chemokines (*p* = 0.008), cytokines (*p* = 0.018), interleukins (*p* = 0.010), macrophage functions (*p* = 0.031), NK-cell functions (*p* = 0.024), and leukocyte functions (*p* = 0.006) were downregulated (Figure 2C).

### 3.3. Hepatic Gene Expression Changes Associated with Fibrosis Progression from F1 to F4 in NAFLD Patients

**Figure 3:**
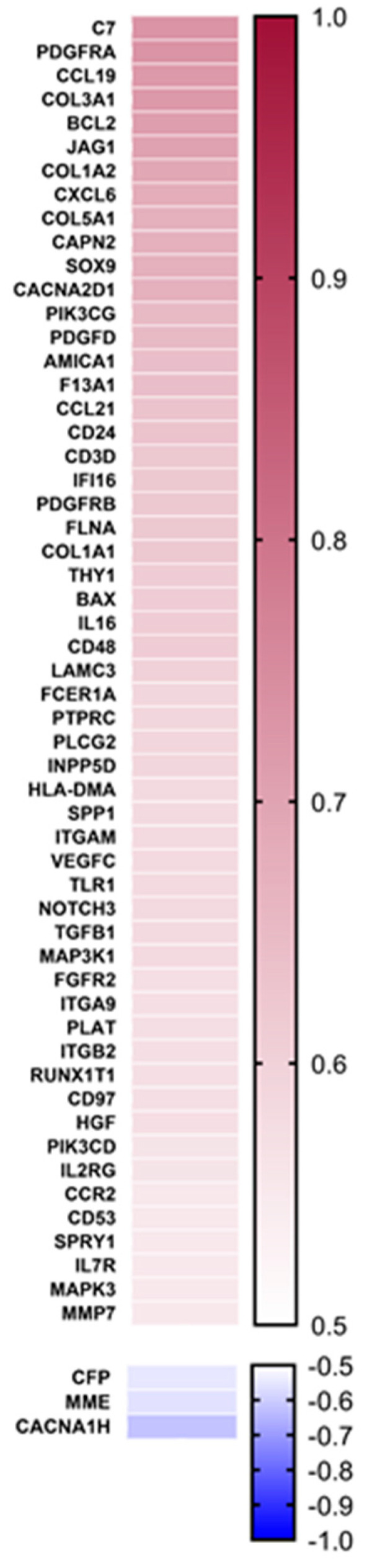
Spearman’s correlation of fibrosis stages F0-F4 and genes from PanCancer Immune Profiling panel and PanCancer Pathways panel. Genes are sorted by correlation coefficient, and coefficient cut-offs of 0.55 and −0.55 were applied.

To identify genes that are associated with the progression of liver fibrosis from F1 to F4, we performed Spearman correlation analyses between the expression levels of all detected genes and fibrosis stages in the 99 NAFLD patients (Figure 3, Supplementary Table S3). As anticipated, a strong positive correlation was observed for collagen type 1 alpha 2 and type III alpha 1 chains (Col1A2, Col3A1) (r = 0.75, *p* < 0.001; r = 0.72, *p* < 0.001). Other genes with strong positive correlations included complement C7 (C7) (r = 0.73, *p* < 0.001); BCL2 apoptosis regulator (BCL2) (r = 0.80, *p* < 0.001); platelet-derived growth factor receptors alpha and beta (PDGFRA, PDGFRB) (r = 0.79, *p* < 0.001; r = 0.71, *p* < 0.001); calcium voltage-gated channel auxiliary subunit alpha2delta 1 (CACNA2D1) (r = 0.75, *p* < 0.001); CCL19 (r = 0.75, *p* < 0.001); jagged canonical notch ligand 1 (JAG1) (r = 0.74, *p* < 0.001); and SRY-box transcription factor 9 (SOX9) (r = 0.74, *p* < 0.001). The strongest negative correlations were observed for MME (r = −0.68, *p* < 0.001); calcium voltage-gated channel subunit alpha1 H (CACNA1H) (r = −0.68, *p* < 0.001); interleukin 10 (IL-10) (r = −0.62, *p* < 0.001); IL-6 receptor (IL-6R) (r = −61, *p* < 0.001); and complement factor properdin (CFP) (r = −0.60, *p* < 0.001). 

### 3.4. Early Gene Expression Changes Predicting Fast Liver Fibrosis Progression: A Pilot Study

**Figure 4:**
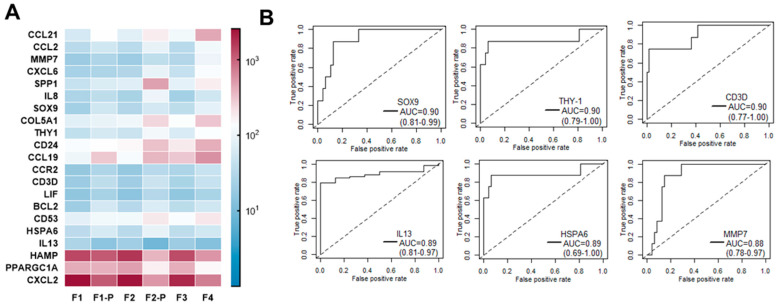
Gene predictors of fast fibrosis progression in NAFLD patients with F1/F2 liver fibrosis. (**A**) Heatmap of genes with significant differential expression in fast progressors (F1-P and F2-P). (**B**) Receiver operating characteristic (ROC) curves for selected genes with highest performance in discriminating fast progressors. The true-positive rate (sensitivity) is plotted as a function of the false-positive rate (100 specificty). Area under the curve (AUC) values as well as 95% lower and upper confidence intervals are shown for SOX9, THY-1, CD3D, IL13, HSPA6, and MMP7.

Using the same two targeted panels (PanCancer Immune Profiling and PanCancer Pathways), we also measured gene expression in liver biopsies from an independent group of eight NAFLD patients with F1 or F2 who progressed rapidly to F3 or F4. Demographic and clinical parameters that significantly differed between these eight patients and NAFLD patients with F1 or F2 among the cohort (n = 62) included older age in progressors (*p* = 0.042); race, with a higher representation of Hispanics (*p* = 0.040); as well as higher AST and ALT levels in progressors, with a median AST value of 63 compared to 30 (*p* < 0.001) and a median ALT value of 57 compared to 44 (*p* = 0.021) (Supplementary Table S1). We compared gene expression in these fast progressors to the 62 F1 or F2 NAFLD patients analyzed above and identified 21 genes with significant expression changes between these two groups (17 higher and 4 lower) (Figure 4A). The strongest differentials were observed in patients with F2 for SPP1 (FC = 6.5, *p* = 0.004); HAMP (FC = −4.5, *p* = 0.005); CXCL2 (FC = −4.5, *p* = 0.004); IL-8 (FC = 3.9, *p* = 0.002); and CXCL6 (FC = 3.2, *p* < 0.001). ROC curves were plotted using the 21 genes with differential expression in fast progressors. SOX9, THY-1, and CD3D had the highest AUC values (0.90, 95% CI = [0.81–0.99]; 0.90, 95% CI= [0.79–1.00]; and 0.90, 95% CI = [0.77–1.00], respectively) followed by IL13 (0.89, 95% CI = [0.81–0.97]); HSPA6 (0.89, 95% CI = [0.69–1.00]); and MMP7 (0.88, 95% CI = [0.78–0.97]) (Figure 4B). Hierarchical clustering analysis using the expression of these six genes clustered the progressors separately from all but three other F1/F2 patients (Supplementary Figure S1). Follow-up fibroscan screening was performed on one of the three subjects clustered with the progressors and remarkably indicated progression to fibrosis F3 after 3.5 years.

### 3.5. mIF Staining for Immune Cell Type Distribution in NAFLD Fibrotic Livers

**Figure 5:**
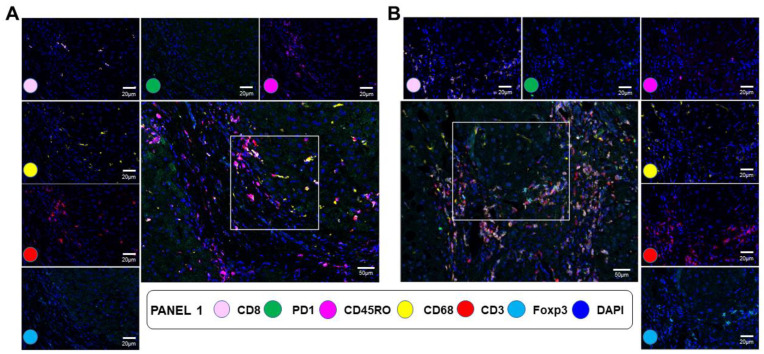
Multiplex IF T-cell panel comparing F1/F2 and F3/F4. This figure includes representative data from F1/F2 (**A**) vs. F3/F4 (**B**) liver biopsies. A composite image showing all markers of the panel, activated simultaneously, is shown in the middle (scale bar: 50 μm). The figure also includes individual composite images showing the CD8-positive expression (pink—opal 540), PD1-positive expression (green—opal 650), CD45RO=positive expression (magenta—opal 570), CD68-positive expression (yellow—opal 520), CD3-positive expression (red—opal 690), and Foxp3-positive expression (cyan—opal 620) of immune cells using inForm^®^ image analysis software, Akoya bioscience (scale bar: 20 μm; Vectra Polaris scanner). The panel also includes a universal biomarker for nuclear detection (blue—DAPI).

We further characterized selected immune cell type changes according to fibrosis severity, using liver biopsies from 22 of the 99 NAFLD patients with an equal distribution of fibrosis scores from F1 to F4. Because of the strong positive correlation between fibrosis severity and the expression of CD3D (r = 0.62, *p* < 0.001); CD3E (r = 0.51, *p* < 0.001); and CD3G (r = 0.47, *p* < 0.001) (Supplementary Table S3) and the strong performance of CD3D in predicting fast liver fibrosis progression in patients with F1/F2 (Figure 4B), we evaluated the distribution of T cells using a mIF panel including antibodies against CD3, CD8, PD1, FOXP3, and CD45RO (Figure 5). This panel also included a target against CD68 to compare the overall distribution of total T cells and total macrophages. In epithelial areas, the number of total CD3^+^ T cells and total CD68^+^ macrophages were comparable, averaging 29.2 cells/mm^2^ and 38.9 cells/mm^2^, respectively. In contrast, fibrotic areas were strongly enriched in CD3^+^ T cells compared to CD68^+^ macrophages (971.0 cells/mm^2^ vs. 194.3 cells/mm^2^, *p* < 0.001). While the number of CD68^+^ macrophages also increased in fibrotic areas, the increase in CD3^+^ T cells was eight-fold higher (*p* < 0.001). Overall, the number of CD3^+^ T cells increased with fibrosis severity from an average of 130.25 cells/mm^2^ in F1/F2 to 400.17 cells/mm^2^ in F3/F4 (FC = 3.1, *p* = 0.008), confirming the gene expression data. Importantly, this increase was progressive, as shown by the strong correlation between fibrosis stage and the total number of CD3^+^ T cells (r = 0.85, *p* = 0.005). The majority of CD3^+^ T cells in stage F3/F4 patients were CD3^+^CD45RO^+^CD8^−^ memory helper T-cells, increasing from 28.51 cells/mm^2^ in F1/F2 to 149.91 cells/mm^2^ in F3/F4 (FC = 5.3, *p* = 0.008; r = 0.88, *p* = 0.002), followed by cytotoxic CD3^+^CD8^+^ T cells, increasing from 38.13/mm^2^ in F1/F2 to 77.60/mm^2^ in F3/F4 (FC = 2.0, *p* = 0.05; r = 0.66, *p* = 0.05). The largest differential increase during fibrosis progression was observed for two regulatory T cell subpopulations positive for FOXP3 and negative for CD8: CD3^+^CD45RO^+^FOXP3^+^CD8^−^ (0.67 cells/mm^2^ in F1/F2 to 9.23 cells/mm^2^ in F3/F4; FC = 13.7, *p* = 0.008; r = 0.77, *p* = 0.014) and CD3^+^CD45RO^−^FOXP3^+^CD8^−^ (1.89 cells/mm^2^ in F1/F2 to 19.78 cells/mm^2^ in F3/F4; FC = 10.5, *p* = 0.016; r = 0.71, *p* = 0.029). A strong progressive increase with fibrosis severity was also observed for CD3^+^CD45RO^+^CD8^+^ effector memory T cells (5.29 cells/mm^2^ in F1/F2 to 24.74 cells/mm^2^ in F3/F4; FC = 4.7, *p* = 0.008; r = 0.91, *p* = 0.001). Finally, antigen-experienced CD3^+^PD-1^+^ T cells were detected at all fibrosis stages (15.15 cells/mm^2^ in F1/F2 and 34.38 cells/mm^2^ in F3/F4) and slightly increased during fibrosis progression but not significantly.

**Figure 6:**
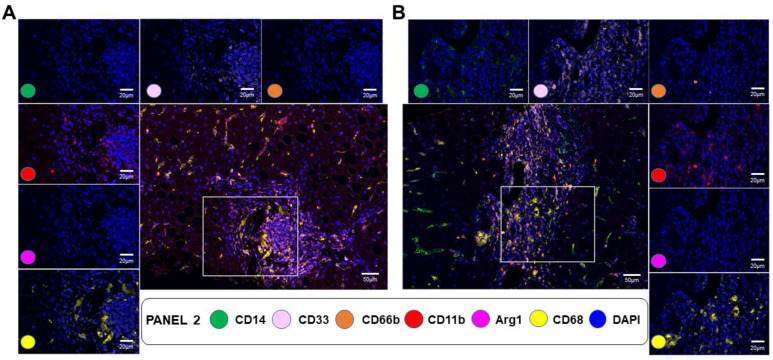
Multiplex IF myeloid cell panel comparing F1/F2 and F3/F4. This figure includes representative data from F1/F2 (**A**) vs. F3/F4 (**B**) liver biopsies. A composite image showing all markers of the panel, activated simultaneously, is shown in the middle (scale bar: 50 μm). The figure also includes individual composite images showing CD14-positive expression (green—opal 650), CD33-positive expression (pink—opal 540), CD66-positive expression (orange—opal 570), CD11-positive expression (red—opal 690), ARG1-positive expression (magenta—opal 570), and CD68-positive expression (yellow—opal 520) of immune cells using inForm^®^ image analysis software, Akoya bioscience (scale bar: 20 μm; Vectra Polaris scanner). The panel also includes a universal biomarker for nuclear detection (blue—DAPI).

As ITGAM/CD11b gene expression strongly correlated with fibrosis stage (r = 0.63, *p* < 0.001) (Supplementary Table S3), we also evaluated CD11b^+^ cell populations using an mIF panel targeting myeloid cells and including antibodies against CD11b, CD68, Arg1, CD66b, CD14, and CD33 (Figure 6). The number of CD68^+^CD11b^+^ Kupffer cells strongly increased with fibrosis severity from an average of 10.88 cells/mm^2^ in F1/F2 to 83.74 cells/mm^2^ in F3/F4 (FC = 7.7, *p* = 0.05). The number of CD66b^+^CD11b^+^ myeloid granulocytic cells and CD66b^+^CD33^+^CD11b^+^ granulocytic MDSCs were not significantly different between different fibrosis stages. Arg1^+^CD68^+^CD11b^+^ myeloid macrophages and monocytic myeloid-derived suppressor cells (M-MDSCs, Arg1^+^CD14^+^CD33^+^CD11b^+^) were largely undetectable in fibrotic areas.

## 4. Discussion

Clinical factors cannot accurately predict the risk of developing cirrhosis in patients with NAFLD. This study aimed to characterize molecular and cellular changes occurring during liver fibrosis progression in NAFLD patients and propose a predictive gene signature for progression to advanced fibrosis. Differentiating subjects at a high risk for advanced liver fibrosis/cirrhosis versus subjects at a low risk would be highly valuable in clinical practice, helping to reduce unnecessary liver biopsies in follow-up visits and increasing surveillance in high-risk subjects. Our molecular and cellular study also has the potential to accelerate drug development for the treatment of liver fibrosis and the prevention of HCC.

While a progressive shift in overall gene expression according to the fibrosis stage was observed from F1 to F4, the strongest difference was observed between F3 and F4, with 162 cirrhosis-associated gene changes identified. Strong correlations with fibrosis progression from F1 to F4 were observed for 91 of these 162 genes. These included CCL21, CCL2, CXCL6, and CCL19. Remarkably, the expression of these four chemokines was also significantly higher in fast-progressor patients, suggesting that serum levels of these four chemokines in NAFLD patients could serve as a predictor of fibrosis progression or as companion biomarkers of clinical trials for NASH therapies. Among them, CCL2 has already been recognized as a multifunctional regulator of liver pathology and an important serum marker of inflammation in NASH and other chronic liver diseases [24,25,26,27]. In mice, the deletion or antibody blockade of CCL2 ameliorated NASH progression and liver fibrosis [28,29,30]. In contrast, the roles in the liver of CCL21 and CCL19, two ligands of CCR7, and CXCL6 remain largely unknown. One study reported that CCL21 expression during chronic hepatitis C is implicated in the recruitment of T lymphocytes and may promote fibrogenesis via the activation of CCR7 on hepatic stellate cells (HSCs) [31]. An expansion of lymphatic endothelial cells with the increased expression of CCL21 and the production of IL13 was reported in NASH [32]. High expression levels of CCL9 were detected in NAFLD patients and increased with NAFLD severity [33]. Finally, mice lacking CCR7 are protected from diet-induced obesity and insulin resistance [34]. Future spatial analyses of CCL21 and CCL19 expression in the context of HSCs and T cells in NASH liver biopsies are warranted. Interestingly, CXCL6 was one of six genes included in a Cirrhosis Risk Score (CRS), a predictive gene signature for cirrhosis in patients with chronic hepatitis B [35]. Other genes in the CRS included CD24 and SOX9, markers of liver progenitor cells. The expression of both CD24 and SOX9 was identified in our study to strongly correlate with liver fibrosis progression and was specifically elevated in fast-progressor NAFLD patients. Expression levels of SOX9 had a particularly strong predictive performance for liver fibrosis progression. We previously reported that miR-21 inhibition reduces liver fibrosis by inducing the apoptosis of CD24^+^ progenitor cells, confirming a major role of these cells in fibrogenesis [36]. It was also reported that CD24 polymorphisms affect the progression of chronic hepatitis B infection [37]. SOX9 expression in biopsies from patients with chronic hepatitis C was found to correlate with fibrosis severity and accurately predicted disease progression toward cirrhosis [38]. In addition, in these patients, levels of SOX9-regulated proteins such as SPP1, another liver progenitor marker, were increased in the serum and correlated with the severity of liver fibrosis [39]. Interestingly, the expression of SPP1 was identified in our study to correlate with fibrosis progression and was specifically elevated in fast-progressor NAFLD patients. Both SPP1 and CD24 were identified in independent studies as crucial genes in NASH [10,40]. Plasma IL-8 levels have also been reported to correlate with SOX9 expression in human liver specimens [41]. Altogether, these results suggest that the SOX9 activation network and the expansion of liver progenitor cells either by increased survival or the dedifferentiation of hepatocytes are common mechanisms of fibrosis progression in NASH and other chronic liver diseases leading to cirrhosis. In agreement with this conclusion, recent studies using total transcriptomics combined with cell-type deconvolution or single-cell RNA sequencing also identified a loss of hepatocytes and increased markers of bipotent hepatocyte/cholangiocyte precursors with advancing fibrosis stage in NAFLD [10,15].

Several other genes not described above were found to be elevated in fast-progressor NAFLD patients. A major example is CCR2. CCR2-expressing macrophages localize to hepatic crown-like structures in NASH livers [42]. While there is no approved liver-specific drug therapy with a proven effect on NASH patients, Cenicriviroc (CVC), a dual inhibitor of CCR2 and CCR5, is among the most advanced substances in clinical development in ongoing phase 2 and 3 studies [43]. CVC is under evaluation for treating liver fibrosis in adults with NASH. The majority of patients on CVC who achieved fibrosis response at year 1 maintained it at year 2, with a greater effect on advanced fibrosis [44,45]. Other examples include THY1, which was also found among 25 differentially expressed genes as fibrosis steatohepatitis progressed through stages F2 to F4 in a large multicenter study [13], and MMP7, whose serum levels were independently associated with clinically significant fibrosis and improving the diagnostic performance of currently available tests in NAFLD patients [46].

The genes most strongly downregulated in F4 compared to F3 included PPARGC1A and HAMP. The expression of both PPARGC1A and HAMP was also significantly lower in fast-progressor NAFLD patients, while it was not negatively correlated with fibrosis progression from F1 to F4, suggesting that reductions in PPARGC1A and HAMP expression are not early events in fibrogenesis but important events in progression to advanced liver fibrosis stages. PPARGC1A plays an important role in mitochondria biogenesis and oxidative stress. Several studies have demonstrated the importance of PPARGC1A in mitochondrial β-oxidation and oxidative stress in the pathogenesis of NAFLD [47,48]. In addition, associations between PPARGC1A polymorphisms and NAFLD risk and severity in hepatic histological features have been reported in both adults and children [49,50,51]. Importantly, PPARGC1A-knockout mice challenged with CCL_4_ or a high-fat diet presented abnormal mitochondrial fission, increased oxidative stress, and more severe liver fibrosis than wild-type mice [52,53]. The HAMP gene is highly expressed in liver and encodes hepcidin, an antimicrobial peptide and key iron regulatory hormone. The overexpression of hepcidin alleviates steatohepatitis and fibrosis in diet-induced NASH [54].

In addition to SOX9, THY-1, CD3D, IL13, HSPA6, and MMP7 demonstrated the best performance in detecting fast-progressor NAFLD patients with F1/F2. A signature composed of these six genes clustered the fast-progressors together and separately from other patients with F1/F2 fibrosis, with the exception of three patients. Remarkably, fibroscan follow-up data available for one of these three patients showed a progression to F3 after 3.5 years. The utility of this six-gene signature in predicting progression to advanced fibrosis should therefore be validated in a large prospective cohort.

We also characterized selected immune-cell-type changes using mIF platforms. In recent years, single-cell RNA sequencing has highlighted the heterogeneity of liver immune cells in healthy patients and those with diseases, including NAFLD [55]. Spatial analysis provides additional layers of spatial heterogeneity information but can therefore improve our understanding of the mechanisms behind the formation of the patterns observed in fibrotic livers. The multiplex T-cell platform showed that fibrotic areas were strongly enriched in CD3^+^ T cells compared to CD68^+^ macrophages, and that while the number of CD68^+^ macrophages also increased with fibrosis severity, the magnitude of the increase in density was stronger for CD3^+^ T cells and progressive from F1 to F4, confirming the gene expression data. The highest density of CD3^+^ T cells in F3/F4 was observed for CD3^+^CD45RO^+^CD8^−^ memory helper T cells. It has been previously reported that the infiltration of CD4^+^ memory T cells contributed to immune microenvironment changes in cirrhosis [56]. Most importantly, among all T-cell subsets analyzed in our panel, the strongest correlation with fibrosis progression was observed for CD3^+^CD45R0^+^ memory T cells, while the largest increase in density between F1/F2 and F3/F4 was observed for regulatory CD3^+^CD45RO^+^FOXP3^+^CD8^−^ and CD3^+^CD45RO^−^FOXP3^+^CD8^−^ T cells. A dual role for regulatory T cells in liver fibrosis was reported, promoting both immunosuppression and the activation of fibrosis [57]. We also used a second mIF panel to better characterize CD11b^+^ cells in liver fibrosis progression and identified a specific increase in the density of CD68^+^CD11b^+^ cells. CD11b is expressed on all macrophages in the liver (the liver’s resident macrophages, commonly named Kupffer cells, and bone-marrow-derived macrophages), and it is generally agreed that cells expressing CD11b and CD68 are Kupffer cells [58]. The dysregulation of Kupffer cells in NASH can increase fibrosis through the production of cytokines such as TGF-beta1 and IL-6 [59]. Kupffer cells, regulatory T cells, and memory T cells should be further characterized in NASH and their exact role in relation to liver fibrosis thoroughly researched.

## 5. Conclusions

Overall, this comprehensive study of liver-fibrosis-associated gene expression and immune cell changes in NAFLD identified gene signatures and cell subsets that strongly correlated with liver fibrosis progression. These should be further characterized for their functional roles as well as predictive performance in prospective longitudinal cohorts.

## Data Availability

The data presented in this study are available on request from the corresponding author.

## Supplementary Materials

The following supporting information can be downloaded at: https://www.mdpi.com/article/10.3390/cancers15112871/s1, Table S1: Demographic and clinical parameters for 99 NAFLD patients and 8 progressors, Table S2: Expression of genes from the PanCancer Immune Profiling panel or the PanCancer Pathways panel found to be upregulated or downregulated in liver biopsies from NAFLD patients with cirrhosis (F4) compared to NAFLD patients with liver fibrosis stage F3; Table S3: Positive (r ≥ 0.40, *p* < 0.05) and negative (r ≤ −0.40, *p* < 0.05) correlations between gene expression levels and fibrosis stages F0–F4, Figure S1: Hierarchical clustering of NAFLD patients with F1/F2 including eight fast progressors (F1P/F2P).
