# Supplementary material for: Cellular and Molecular Mechanisms of Liver Fibrosis in Patients with NAFLD
Source: Cancers (Basel). 2023 May 23;15(11):2871. doi: 10.3390/cancers15112871 (PMC10252068; doi:10.3390/cancers15112871)
Supplement: Supplementary file 1 [file cancers-15-02871-s001.zip › Figure S1.pdf]

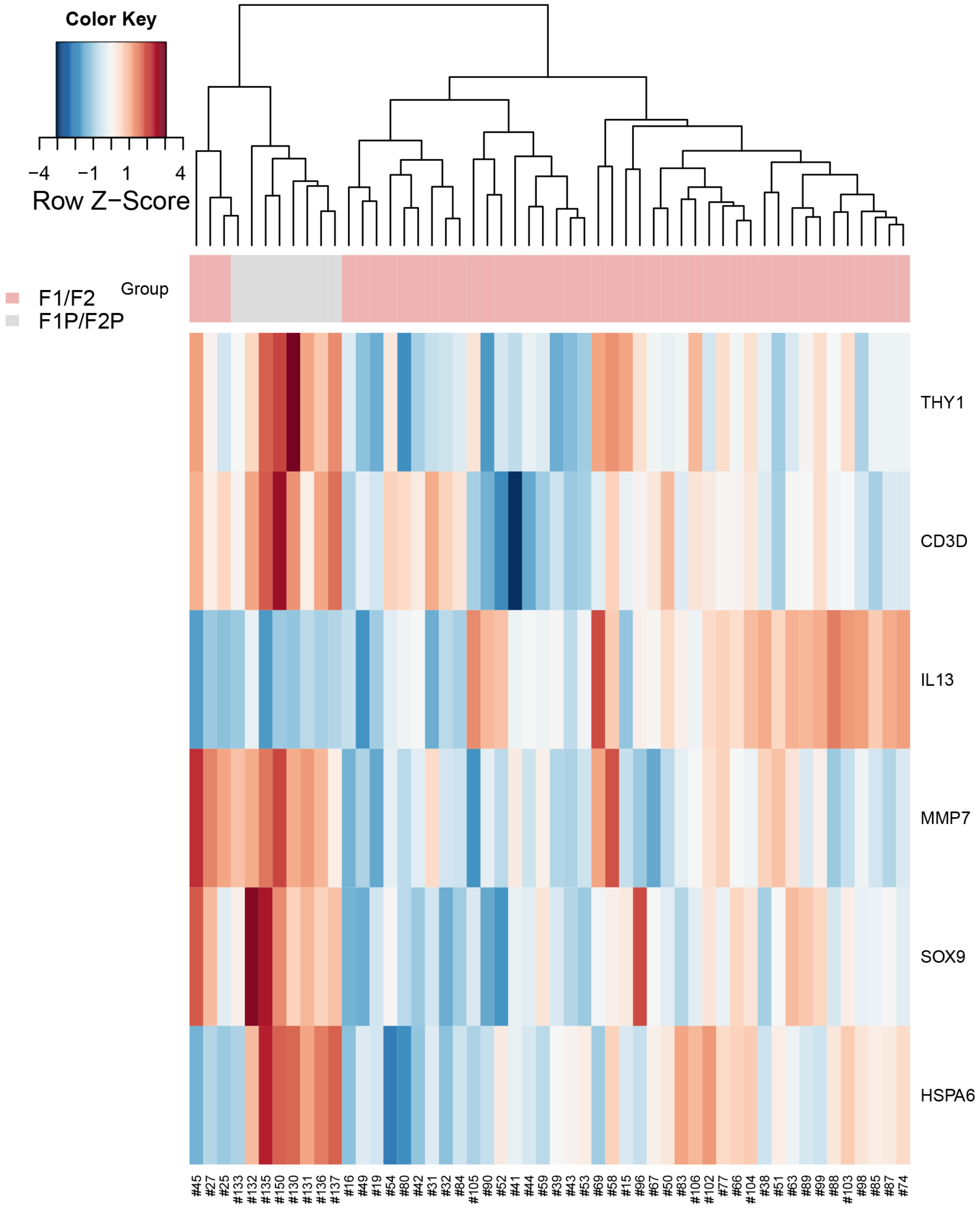


**Supplementary Figure S1. Hierarchical clustering of NAFLD patients with F1/F2 including 8 fast progressors (F1P/F2P).** The six genes with the highest ROC performances in discriminating fast progressors were used to generate a heatmap using log10-transformed gene expression data. Color key represents row Z-scores.
