# Supplementary material for: Cellular and Molecular Mechanisms of Liver Fibrosis in Patients with NAFLD
Source: Cancers (Basel). 2023 May 23;15(11):2871. doi: 10.3390/cancers15112871 (PMC10252068; doi:10.3390/cancers15112871)
Supplement: Supplementary file 1 [file cancers-15-02871-s001.zip › Table S1.pdf]

Supplementary Table S1: Demographic and clinical parameters for 99 NAFLD patients including 62 F1/F2 patients, and eight progressors F1P/F2P. Data are displayed as n (%) or mean (range)-median. AFP: alpha-fetoprotein; ALB: albumin; ALT: alanine aminotransferase; AST: aspartate aminotransferase; BG: blood glucose; BMI: body mass index; HbA1c: hemoglobin A1c; NAS: NAFLD activity score; PLT: platelets; PT: prothrombin time. Significant p-values between the two groups of patients are shown. P-values between F1/F2 (n=62) and F1P/F2P (n=8) are shown in the last column when significant (<0.05).

**n=107**	**NAFLD F1-F4 (n=99)**	**F1/F2 (n=62)**	**F1P/F2P (n=8)**	**p-values**
Male	33 (33%)	16 (26%)	3 (38%)	
Age	48 (25-75) - 50	48 (25-61) - 49	55 (46-63) - 55	0.042
Diabetes II	50 (51%)	29 (47%)	4 (50%)	
BMI	42.2 (17.0-68.7) - 42.4	45.2 (2.42-68.7) - 44.6	30.9 (26.2-33.8) - 31.8	<0.001
Obesity (BMI≥30)	86 (87%)	59 (98%)	3 (75%)	
**Race**				0.040
American Indian	1 (1%)	1 (2%)		
Black	7 (7%)	6 (10%)		
Hispanic	18 (18%)	11 (18%)	5 (63%)	
Caucasian	73 (74%)	44 (71%)	3 (38%)	
**Steatosis**				0.018
0	3 (3%)	-		
1	36 (36%)	21 (34%)	5 (62%)	
2	43 (43%)	28 (45%)	3 (38%)	
3	17 (17%)	13 (21%)		
**Fibrosis Stage**				
1	32 (32%)	32 (52%)	3 (38%)	
2	30 (30%)	30 (48%)	5 (63%)	
3	15 (15%)			
4	22 (22%)			
**Inflammation Score**				
0	4 (4%)	4 (6%)		
1	48 (48%)	35 (56%)	5 (63%)	
2	43 (43%)	22 (35%)	3 (38%)	
3	4 (4%)	1 (2%)		
**Ballooning**				
0	35 (35%)	24 (39%)	2 (25%)	
1	50 (51%)	34 (55%)	5 (63%)	
2	14 (14%)	4 (6%)	1(13%)	
**NAS**				

1	5 (5%)	3 (5%)		
2	15 (15%)	8 (13%)	1 (13%)	
3	16 (16%)	14 (23%)	3 (38%)	
4	20 (20%)	14 (23%)	3 (38%)	
5	28 (28%)	17 (27%)	1 (13%)	
6	11 (11%)	4 (6%)		
7	4 (4%)	2 (3%)		
**Laboratory tests**				
HgA1C	6.9 (4.8-12.9) - 6.4	6.7 (5.0-11.7) - 6.3		
PT (s)	13.8 (11.0-18.0) - 13.7	13.0 (11.0-15.9) - 12.7	15.6 (13.4-26.8) - 13.4	0.030
ALB (ng/mL)	4.1 (2.1-5.2) - 4.1	4.2 (3.3-4.9) - 4.2	4.1 (3.8-4.5) - 4.0	
PLT ($x10^9/L$)	234 (4.5-433) - 235	261.7 (4.5-398) - 262	208.1 (105-252) - 226	0.049
AST (U/L)	53.4 (17-582) - 35	36.5 (17-157) - 30	120.6 (26-482) - 63	<0.001
ALT (U/L)	57.2 (18-252) - 46	52.9 (18-252) - 44	90.3 (23-224) - 57	0.021
BG (mg/dL)	131.3 (62-370) - 115	127.2 (64-283) - 118	114.2 (61-162) - 103	
