# Supplementary material for: Cellular and Molecular Mechanisms of Liver Fibrosis in Patients with NAFLD
Source: Cancers (Basel). 2023 May 23;15(11):2871. doi: 10.3390/cancers15112871 (PMC10252068; doi:10.3390/cancers15112871)
Supplement: Supplementary file 1 [file cancers-15-02871-s001.zip › Table S2.pdf]

Supplementary Table S2: Expression of genes from the PanCancer Immune Profiling panel or the PanCancer Pathways panel, found to be upregulated or downregulated in liver biopsies from NAFLD patients with cirrhosis (F4) compared to NAFLD patients with liver fibrosis stage F3. The following data are shown: mean of expression in F3, mean of expression in F4, fold change (FC) F4 vs F3, p-value, adjusted p-value (q-value) by Benjamini-Hochberg method.

**Immune Profiling panel**	F3 mean	F4 mean	FC	p-value	q-value
**Upregulated genes**					
CCL21	110.23	436.61	3.96	<0.001	<0.001
ANXA1	85.84	294.83	3.43	<0.001	<0.001
CCL2	34.09	109.44	3.21	<0.001	<0.001
F13A1	44.48	138.77	3.12	<0.001	<0.001
CXCL6	42.98	128.51	2.99	<0.001	<0.001
SPP1	64.79	184.81	2.85	<0.001	<0.001
LGALS3	84.37	219.72	2.60	<0.001	<0.001
ITGB4	23.56	58.41	2.48	0.003	0.020
IL8	22.61	55.81	2.47	0.001	0.009
C7	710.98	1691.30	2.38	<0.001	<0.001
PDGFRB	123.75	272.25	2.20	<0.001	<0.001
THY1	64.64	140.70	2.18	<0.001	<0.001
EPCAM	35.02	73.85	2.11	<0.001	<0.001
COL3A1	973.54	2037.80	2.09	<0.001	<0.001
CD24	195.94	408.19	2.08	<0.001	<0.001
CCL19	294.34	574.43	1.95	0.009	0.042
CXCL12	600.29	1163.38	1.94	0.002	0.015
SYK	26.95	47.78	1.77	<0.001	<0.001
FCER1A	31.58	55.64	1.76	0.006	0.033
CCR2	26.42	46.05	1.74	0.003	0.020
IL2RG	45.31	78.84	1.74	<0.001	<0.001
CD3D	25.66	44.60	1.74	<0.001	<0.001
ENG	230.11	398.14	1.73	<0.001	<0.001
PLAU	23.87	40.82	1.71	<0.001	<0.001
HLA-DPB1	494.54	841.11	1.70	0.009	0.042
PECAM1	152.95	258.13	1.69	0.003	0.020
HLA-DRA	968.08	1623.61	1.68	0.030	0.098
TFRC	112.28	187.20	1.67	0.001	0.009
IFI16	71.53	118.12	1.65	<0.001	<0.001
IFITM1	209.29	339.88	1.62	0.006	0.033
LY9	22.42	36.14	1.61	0.001	0.009
CD96	34.70	55.87	1.61	0.001	0.009
ITGA2	13.17	21.13	1.60	0.003	0.020

ITGA6	52.27	82.71	1.58	<0.001	<0.001
CSF1	25.33	39.99	1.58	0.004	0.025
MFGE8	65.23	102.84	1.58	0.003	0.020
GZMA	36.43	57.28	1.57	0.006	0.033
PTPRC	89.72	140.89	1.57	0.008	0.041
HLA-DPA1	483.32	757.71	1.57	0.011	0.048
AMICA1	108.66	170.01	1.56	<0.001	<0.001
CD9	100.00	155.17	1.55	0.009	0.042
CD53	129.73	199.54	1.54	0.006	0.033
CD3G	10.75	16.52	1.54	<0.001	<0.001
MEF2C	52.62	80.75	1.53	0.002	0.015
FCGR2A	72.39	110.99	1.53	0.004	0.025
LTB	48.14	73.67	1.53	0.020	0.075
TNFRSF11B	21.00	32.13	1.53	0.010	0.045
IRF1	48.18	73.51	1.53	<0.001	<0.001
ITGB1	832.56	1262.62	1.52	0.001	0.009
GZMK	33.59	50.91	1.52	0.015	0.060
ITGB2	86.69	131.32	1.51	<0.001	<0.001
SMAD2	74.22	111.74	1.51	0.001	0.009
**Downregulated genes**					
MAP2K1	230.45	154.37	-1.50	0.009	0.042
LILRA5	23.75	15.90	-1.50	0.002	0.015
PTGDR2	11.84	7.83	-1.51	0.004	0.025
SYCP1	15.02	9.90	-1.52	0.009	0.042
MAGEA3	18.35	12.04	-1.52	0.020	0.075
CD163	330.96	216.46	-1.53	<0.001	0.000
EBI3	20.47	13.35	-1.53	0.045	0.125
XCR1	17.60	11.45	-1.54	0.014	0.059
IL22RA2	18.75	12.18	-1.54	0.036	0.111
IL13	19.66	12.76	-1.54	0.022	0.080
STAT3	1301.81	843.32	-1.54	0.002	0.015
C6	2270.26	1470.17	-1.54	<0.001	<0.001
KLRF1	17.18	11.07	-1.55	0.003	0.020
KLRD1	30.36	19.51	-1.56	0.001	0.009
IL4	20.48	13.14	-1.56	0.002	0.015
IL26	14.44	9.19	-1.57	0.039	0.115
IL1RAP	284.81	181.06	-1.57	0.005	0.030
CEBPB	518.61	329.52	-1.57	0.002	0.015
MERTK	214.62	136.16	-1.58	0.001	0.009
CMA1	12.41	7.84	-1.58	0.049	0.135
IL1RL1	29.21	18.44	-1.58	0.009	0.042
ARG2	25.79	16.10	-1.60	0.002	0.015
RAG1	17.60	10.98	-1.60	0.003	0.020
TPTE	15.07	9.39	-1.61	0.022	0.080

ARG1	2213.43	1366.97	-1.62	0.005	0.030
BLK	24.75	15.28	-1.62	0.039	0.115
CTCFL	17.84	10.89	-1.64	0.010	0.045
SEMG1	18.12	11.04	-1.64	0.001	0.009
IL24	17.36	10.55	-1.65	0.039	0.115
CD1A	17.68	10.59	-1.67	0.033	0.105
IL6R	159.35	93.00	-1.71	<0.001	<0.001
IL12A	17.86	10.42	-1.71	0.003	0.020
CCL23	14.02	8.15	-1.72	<0.001	<0.001
IL13RA2	29.34	17.00	-1.73	0.001	0.009
TMEFF2	21.13	12.14	-1.74	0.015	0.060
IL10	20.04	11.43	-1.75	0.009	0.042
IFNA7	19.29	10.91	-1.77	0.009	0.042
C4B	9701.57	5361.52	-1.81	0.010	0.045
ELK1	91.56	50.48	-1.81	0.015	0.060
CXCR1	26.02	14.34	-1.81	0.030	0.098
CFP	98.14	54.02	-1.82	<0.001	<0.001
PRKCE	78.49	43.12	-1.82	0.001	0.009
IFNL1	58.11	31.12	-1.87	0.039	0.115
IGLL1	16.56	8.82	-1.88	0.009	0.042
IL27	34.16	17.77	-1.92	0.020	0.075
MARCO	330.76	170.95	-1.93	0.001	0.009
IL1RN	77.52	39.69	-1.95	0.001	0.009
MAGEC2	20.00	10.07	-1.99	0.028	0.095
OSM	19.67	9.83	-2.00	0.001	0.009
CRP	3123.55	1552.18	-2.01	0.010	0.045
MAGEC1	18.55	9.20	-2.02	0.018	0.069
CCL7	19.45	9.45	-2.06	0.002	0.015
TAL1	32.63	15.48	-2.11	0.002	0.015
HAMP	1305.42	539.84	-2.42	0.007	0.037
MME	140.88	56.74	-2.48	<0.001	<0.001
CXCL2	1956.85	726.00	-2.70	0.001	0.009
RORA	555.12	202.05	-2.75	<0.001	<0.001

**Pathways panel**	F3 mean	F4 mean	FC	p-value	q-value
**Upregulated genes**					
MMP7	30.48	96.65	3.17	0.001	0.013
IL8	30.20	82.42	2.73	0.004	0.036
ITGB4	20.53	52.60	2.56	0.010	0.069
LAMC2	6.42	15.68	2.44	<0.001	<0.001

PLAT	50.49	118.85	2.35	<0.001	<0.001
SOX9	37.61	85.63	2.28	<0.001	<0.001
COL1A1	160.68	356.65	2.22	0.003	0.030
COL5A1	137.78	303.05	2.20	0.004	0.036
COL1A2	110.62	239.70	2.17	<0.001	<0.001
ITGB8	22.01	46.97	2.13	0.002	0.021
NOTCH3	67.37	142.74	2.12	0.001	0.013
PDGFRB	98.65	208.68	2.12	0.001	0.013
COL11A1	3.66	7.70	2.10	0.035	0.142
SPP1	83.58	175.13	2.10	0.001	0.013
ANGPT1	12.52	26.04	2.08	<0.001	<0.001
PDGFD	19.40	40.10	2.07	0.002	0.021
FLNA	223.07	454.90	2.04	0.001	0.013
JAG1	52.12	103.07	1.98	<0.001	<0.001
CCND2	31.77	62.76	1.98	0.006	0.045
CREB3L1	9.74	18.32	1.88	0.002	0.021
LEF1	8.28	15.56	1.88	<0.001	<0.001
PDGFRA	150.54	276.84	1.84	<0.001	<0.001
SFRP4	8.09	14.70	1.82	0.014	0.080
COL3A1	989.28	1794.08	1.81	0.004	0.036
PROM1	22.67	39.16	1.73	<0.001	<0.001
HGF	159.73	274.93	1.72	0.001	0.013
PLAU	22.38	38.25	1.71	<0.001	<0.001
CDKN2C	17.88	30.37	1.70	<0.001	<0.001
ITGA6	66.80	111.30	1.67	0.002	0.021
STMN1	38.40	63.89	1.66	0.012	0.071
CAPN2	173.43	284.87	1.64	<0.001	<0.001
ITGA2	14.12	22.99	1.63	0.012	0.071
HSPA2	20.49	32.68	1.59	0.004	0.036
LIF	16.17	25.56	1.58	0.025	0.114
RASGRP1	15.63	24.64	1.58	0.004	0.036
GAS1	19.82	30.99	1.56	0.023	0.111
NGF	11.16	17.44	1.56	0.001	0.013
PBX1	81.72	126.88	1.55	0.002	0.021
RUNX1T1	31.51	48.56	1.54	<0.001	<0.001
BCL2	35.71	55.02	1.54	0.001	0.013
ITGA3	37.84	58.26	1.54	0.002	0.021
CACNA2D1	38.72	59.45	1.54	0.001	0.013
CDKN1C	37.39	57.30	1.53	0.006	0.045
JAK3	32.29	49.34	1.53	<0.001	<0.001
HSPA6	37.54	56.87	1.52	0.017	0.090
MCM5	17.52	26.28	1.50	0.001	0.013

**Downregulated genes**					
TSPAN7	98.93	65.01	-1.52	<0.001	<0.001
LRP2	11.03	7.13	-1.55	0.012	0.071
SGK2	181.45	117.01	-1.55	0.021	0.106
MAP2K1	221.17	142.09	-1.56	0.014	0.080
EGFR	451.14	286.56	-1.57	<0.001	<0.001
IL6R	180.96	114.71	-1.58	0.002	0.021
IL1RAP	254.57	160.89	-1.58	0.006	0.045
PLA2G10	5.93	3.73	-1.59	0.030	0.129
IL13RA2	24.20	14.40	-1.68	0.001	0.013
GHR	779.84	449.70	-1.73	<0.001	<0.001
RET	22.98	12.17	-1.89	0.001	0.013
CACNA1H	166.99	85.07	-1.96	<0.001	<0.001
NR4A1	31.41	15.68	-2.00	0.041	0.158
PCK1	1322.90	637.19	-2.08	0.049	0.182
DDIT4	279.75	131.45	-2.13	0.004	0.036
ZBTB16	441.10	202.42	-2.18	0.005	0.042
GADD45B	410.65	161.88	-2.54	0.030	0.129
PPARGC1A	406.29	160.05	-2.54	0.005	0.042
GADD45G	244.96	57.13	-4.29	0.011	0.071
