# Supplementary material for: Cellular and Molecular Mechanisms of Liver Fibrosis in Patients with NAFLD
Source: Cancers (Basel). 2023 May 23;15(11):2871. doi: 10.3390/cancers15112871 (PMC10252068; doi:10.3390/cancers15112871)
Supplement: Supplementary file 1 [file cancers-15-02871-s001.zip › Table S3.pdf]

Supplementary Table S3: Positive ($r \geq 0.40$, $p<0.05$) and negative ($r \leq -0.40$, $p<0.05$) correlations between gene expression levels and fibrosis stages F0-F4. Spearman correlation values (r) with p-values are shown.

Genes	r	p-value
**Positive correlations**		
C7	0.73	<0.001
PDGFRA	0.73	<0.001
CCL19	0.72	<0.001
COL3A1	0.72	<0.001
BCL2	0.71	<0.001
JAG1	0.70	<0.001
COL1A2	0.69	<0.001
CXCL6	0.68	<0.001
COL5A1	0.67	<0.001
CAPN2	0.67	<0.001
SOX9	0.67	<0.001
CACNA2D1	0.67	<0.001
PIK3CG	0.65	<0.001
PDGFD	0.65	<0.001
AMICA1	0.64	<0.001
F13A1	0.64	<0.001
CCL21	0.63	<0.001
CD24	0.63	<0.001
CD3D	0.62	<0.001
IFI16	0.62	<0.001
PDGFRB	0.62	<0.001
FLNA	0.62	<0.001
COL1A1	0.62	<0.001
THY1	0.61	<0.001
BAX	0.61	<0.001
IL16	0.61	<0.001
CD48	0.61	<0.001
LAMC3	0.60	<0.001
FCER1A	0.59	<0.001
PTPRC	0.59	<0.001
PLCG2	0.59	<0.001
INPP5D	0.59	<0.001
HLA-DMA	0.58	<0.001
SPP1	0.58	<0.001
ITGAM	0.58	<0.001
VEGFC	0.58	<0.001

TLR1	0.58	<0.001
NOTCH3	0.58	<0.001
TGFB1	0.58	<0.001
MAP3K1	0.58	<0.001
FGFR2	0.57	<0.001
ITGA9	0.57	<0.001
PLAT	0.57	<0.001
ITGB2	0.57	<0.001
RUNX1T1	0.57	<0.001
CD97	0.57	<0.001
HGF	0.57	<0.001
PIK3CD	0.56	<0.001
IL2RG	0.56	<0.001
CCR2	0.55	<0.001
CD53	0.55	<0.001
SPRY1	0.55	<0.001
IL7R	0.55	<0.001
MAPK3	0.55	<0.001
MMP7	0.55	<0.001
ANXA1	0.54	<0.001
CD58	0.54	<0.001
DAXX	0.54	<0.001
CASP8	0.54	<0.001
HLA-DPB1	0.54	<0.001
GPC4	0.54	<0.001
HLA-DPA1	0.54	<0.001
GZMA	0.53	<0.001
TNFSF12	0.53	<0.001
COL5A2	0.53	<0.001
ITGAX	0.53	<0.001
NCF4	0.53	<0.001
LGALS3	0.53	<0.001
GZMK	0.53	<0.001
JAK3	0.52	<0.001
JAM3	0.52	<0.001
MFGE8	0.52	<0.001
RELB	0.52	<0.001
SELPLG	0.52	<0.001
LY9	0.52	<0.001
CD59	0.51	<0.001
CD3E	0.51	<0.001
CXCL12	0.51	<0.001
IL32	0.51	<0.001
PECAM1	0.51	<0.001

IL10RA	0.50	<0.001
ITGA3	0.50	<0.001
CD27	0.50	<0.001
WNT4	0.50	<0.001
ITGA6	0.50	<0.001
PLA2G4C	0.50	<0.001
SMARCA4	0.50	<0.001
PBX1	0.49	<0.001
TPSAB1	0.49	<0.001
CCND2	0.49	<0.001
HLA-DMB	0.49	<0.001
NTRK2	0.49	<0.001
FAS	0.49	<0.001
SMAD2	0.49	<0.001
CASP1	0.49	<0.001
CD63	0.48	<0.001
GAS1	0.48	<0.001
DDB2	0.48	<0.001
ANGPT1	0.48	<0.001
ITGB8	0.48	<0.001
CD96	0.48	<0.001
XRCC4	0.48	<0.001
PLAU	0.47	<0.001
RASGRP1	0.47	<0.001
KLRB1	0.47	<0.001
EPCAM	0.47	<0.001
CCL5	0.47	<0.001
ITGB1	0.47	<0.001
CARD11	0.47	<0.001
CD3G	0.47	<0.001
MEF2C	0.47	<0.001
DNMT1	0.46	<0.001
CDKN2C	0.46	<0.001
SHC1	0.46	<0.001
SYK	0.45	<0.001
AKT3	0.45	<0.001
JAG2	0.45	<0.001
ABCB1	0.45	<0.001
IKBKE	0.45	<0.001
CDKN1C	0.45	<0.001
HLA-DRA	0.45	<0.001
CD44	0.45	<0.001
FUT8	0.45	<0.001
CREB3L1	0.44	<0.001

LEF1	0.44	<0.001
FUBP1	0.44	<0.001
PRKAA2	0.44	<0.001
ENG	0.44	<0.001
PRKCD	0.44	<0.001
CD84	0.44	<0.001
MDM2	0.44	<0.001
RAC2	0.43	<0.001
IL8	0.43	<0.001
NFATC1	0.43	<0.001
ETS1	0.43	<0.001
WHSC1L1	0.43	<0.001
CCL2	0.42	<0.001
NGFR	0.42	<0.001
CD74	0.42	<0.001
LTBP1	0.42	<0.001
PROM1	0.42	<0.001
ABL1	0.42	<0.001
CCR5	0.42	<0.001
GNG12	0.42	<0.001
LY86	0.42	<0.001
CCND1	0.42	<0.001
IL7	0.41	<0.001
LYN	0.41	<0.001
CLEC7A	0.41	<0.001
FCGR2A	0.41	<0.001
COL4A4	0.41	<0.001
CXCL10	0.41	<0.001
HSPA6	0.41	<0.001
CXCR6	0.41	<0.001
SPA17	0.40	<0.001
LTB	0.40	<0.001
RB1	0.40	<0.001
MAPK10	0.40	<0.001
ITGA4	0.40	<0.001
CSF1	0.40	<0.001
CD47	0.40	<0.001
ITGB4	0.40	<0.001
COL4A3	0.40	<0.001
**Negative correlations**		
CACNA1H	-0.62	<0.001
MME	-0.56	<0.001
CFP	-0.55	<0.001
C6	-0.53	<0.001

MET	-0.53	<0.001
MASP2	-0.52	<0.001
IL6R	-0.52	<0.001
F12	-0.51	<0.001
ECSIT	-0.50	<0.001
EIF4EBP1	-0.50	<0.001
CLU	-0.48	<0.001
TSPAN7	-0.46	<0.001
C5	-0.46	<0.001
CD160	-0.46	<0.001
CREBBP	-0.45	<0.001
UBB	-0.45	<0.001
CDC14B	-0.45	<0.001
IL10	-0.45	<0.001
RAC3	-0.44	<0.001
AMER1	-0.44	<0.001
ALKBH2	-0.44	<0.001
CFD	-0.44	<0.001
CCL23	-0.44	<0.001
HNF1A	-0.44	<0.001
AR	-0.43	<0.001
POLR2D	-0.43	<0.001
MASP1	-0.42	<0.001
IL1RAP	-0.42	<0.001
PIK3R1	-0.42	<0.001
CXCL2	-0.41	<0.001
IL1RN	-0.41	<0.001
C4B	-0.41	<0.001
CCL7	-0.41	<0.001
TPTE	-0.41	<0.001
PRKCE	-0.40	<0.001
IRF7	-0.40	<0.001
MAPK8	-0.40	<0.001
RORC	-0.40	<0.001
